# Pandemic *Vibrio cholerae* and the Environmental Reservoir Hypothesis—Outstanding Questions Central to Global Cholera Control

**DOI:** 10.1111/1462-2920.70302

**Published:** 2026-04-30

**Authors:** Azfar D. Hossain, William P. Robins, John J. Mekalanos, Louise C. Ivers

**Affiliations:** ^1^ Department of Medicine Massachusetts General Hospital Boston Massachusetts USA; ^2^ Center for Global Health Massachusetts General Hospital Boston Massachusetts USA; ^3^ Department of Microbiology Harvard Medical School Boston Massachusetts USA; ^4^ Department of Global Health and Social Medicine Harvard Medical School Boston Massachusetts USA; ^5^ Harvard Global Health Institute Cambridge Massachusetts USA

## Abstract

Despite global cholera cases rising to more than 560,000 in 2024, there remains no consensus on a fundamental aspect of cholera epidemiology: whether pandemic *Vibrio cholerae* forms long‐term environmental reservoirs which become the source of future outbreaks. This knowledge gap prevents optimal disease forecasting, resource allocation and outbreak response in a variety of at‐risk settings, from places experiencing annual cholera outbreaks (such as the Democratic Republic of the Congo) to those where the disease seemingly reappears after an absence (such as Haiti). Here, we provide a new framework on links between pandemic cholera and ecological parameters, demonstrating a complex and perhaps time‐limited relationship between pathogen and environment. The framework in turn illustrates several outstanding questions which can be addressed to better understand whether, when and how environmental reservoirs perpetuate cholera, thereby helping to advance global cholera control.

## Graphical abstract

Whether, when and for how long pandemic *Vibrio cholerae* forms long‐term environmental reservoirs remains uncertain. We propose a new framework to evaluate possible mechanisms by which the pathogen might experience reservoir formation versus environmental elimination. Further understanding of these mechanisms can in turn inform public health efforts against cholera.

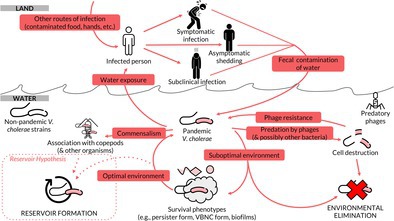

## 1 Introduction

A new outbreak of cholera was declared in September 2022 by Haiti's Ministry of Health when culture‐confirmed cases were identified for the first time in the country since 2019. Within 2 months, this new outbreak had resulted in nearly 12,000 suspected cases of cholera. Genomic studies confirmed *Vibrio cholerae* strains detected in this outbreak were essentially identical to those previously circulating in 2010—different by no more than 53 single nucleotide polymorphisms—making it most likely that cholera had never been eliminated from the country, as some had thought.

Cholera's reemergence in Haiti highlights an important gap in knowledge on a fundamental characteristic of pandemic *V. cholerae* , the subgroup of O1 serogroup bacteria which are the only strains responsible for widespread human disease. There is no consensus on whether or not pandemic *V. cholerae* persists as a reservoir in the environment in the absence of ongoing faecal contamination from humans, which can be due to either outbreaks of disease or asymptomatic or minimally symptomatic bacterial shedding. In other words, a key knowledge gap is whether or not environmental reservoirs of pandemic *V. cholerae* persist in the absence of human infection and disease nearby, later becoming the source of ‘new’ outbreaks. The issue is certainly not limited to Haiti or a new concern, with analyses demonstrating its applicability to cholera‐affected regions around the world for decades (Miller et al. 1985; Domman et al. 2017; Weill et al. 2017), yet the lack of major progress on cholera—with case counts rising in recent years to over 560,000 cases in 2024—calls for renewed attention to the question (World Health Organization 2025).

Ambiguity around whether pandemic *V. cholerae* forms environmental reservoirs limits our understanding of its epidemiology in places where cholera outbreaks appear after extended absence (such as Haiti), recurs sporadically (such as Cameroon) and occurs more frequently (such as the Democratic Republic of the Congo) (World Health Organization 2025); limits the utility of cholera outbreak prediction models; and constrains our understanding of the causal relationship between weather events, climate factors and endemic and epidemic cholera (Chaguza et al. 2024). Cholera outbreak forecasting is essential to inform priority allocation of resources—including cholera vaccines and rapid diagnostic tests as well as prioritisation for water and sanitation infrastructure.

## 2 The Environmental Reservoir Hypothesis

The graphical abstract illustrates known and hypothesized links between pandemic *V. cholerae* and key environmental factors demonstrating a complex, and perhaps time‐limited, relationship with the environment. Studies have revealed multiple plausible mechanisms by which pandemic *V. cholerae* may establish long‐term environmental reservoirs, which we call the ‘environmental reservoir hypothesis’. These include the bacteria's ability to adopt more resilient ‘survivor’ phenotypes in response to environmental stressors, such as a viable‐but‐non‐culturable (VBNC) state (a state similar to spore formation that confers protection against unfavourable ecosystems), a ‘persister’ state (a small bacterial phenotype which may facilitate survival in nutrient‐limited settings for more than 700 days) and protective biofilms (Colwell 1996; Jubair et al. 2012); its commensal relationships with plankton known as copepods, in which increased copepod counts have been associated with increased counts of environmental *V. cholerae* (Colwell 1996) and its ability to develop resistance to predatory phages (Faruque et al. 2005). The environmental reservoir hypothesis is further supported by seasonal trends from South Asia—where cholera cases predictably increase with increasing water temperatures—and observations that other *Vibrio* species appear to persist in environments indefinitely and are climate‐sensitive (Colwell 1996; Archer et al. 2023). These and other data have lent support to various iterations of the ‘cholera paradigm’, which posits *V. cholerae* is a permanent resident of many aquatic ecosystems and its epidemiology is therefore foundationally influenced by changes to environmental parameters (Colwell 1996).

However, a growing number of studies raise doubts about the existence of environmental reservoirs for pandemic *V. cholerae* and the role of the environment in cholera transmission. Genomic studies from the Americas, Africa and specific countries including Argentina and Malawi observed pandemic *V. cholerae* strains responsible for human outbreaks were distinct from *V. cholerae* strains typically found in local water sources and had clear genetic similarities to pandemic strains from South Asia, suggesting outbreaks in these places resulted from travel‐related importation and not emergence of disease from the local environment (Domman et al. 2017; Weill et al. 2017; Chaguza et al. 2024; Dorman et al. 2020). Across 14 Latin American countries from 1974 to 2014, longitudinal environmental sampling before, during and after cholera outbreaks found no evidence of pandemic *V. cholerae* strains forming long‐term reservoirs (Domman et al. 2017). Reasons for lack of environmental reservoirs are not fully understood but may relate to suboptimal environmental conditions, predatory phages and other factors all leading to environmental elimination over time. Simultaneously (see graphical abstract), evidence increasingly supports ‘inapparent infection’—that is, asymptomatic (no symptoms) or subclinical (only minor symptoms, not severe enough to lead to care‐seeking behaviour) infection with pandemic *V. cholerae* —as an alternative explanation for why cholera could seemingly recur in a place even after years of no detected cases (Hegde et al. 2024). One 2024 study from Bangladesh observed that among more than 3000 people with serologic evidence of exposure to *V. cholerae* , more than 99% had few or no symptoms, suggesting that inapparent infection—rather than environmental reservoirs—may play a much larger role in cholera epidemiology than previously presumed (Hegde et al. 2024). Especially important is the understanding of whether or not transmissibility is reduced among these individuals compared to more symptomatic cases.

## 3 Conclusion

Addressing these contradictory observations about environmental reservoirs is key to improving global cholera control strategies. Significant clarity would come from answering two outstanding questions. Firstly, additional environmental surveillance studies employing genomic analyses in places experiencing cholera could answer whether, and for how long, pandemic *V. cholerae* can be detected in environmental sources, across the spectrum of human expression of infection and disease, and including inter‐outbreak periods. Because cross‐sectional environmental studies, like those from Haiti, have limitations—as positive environmental samples may reflect either reservoir formation or ongoing human faecal contamination—these investigations should be longitudinal and also include evaluation for other organisms that may affect *V. cholerae* 's viability such as viruses acting on *V. cholerae* (vibriophages) (Rubin et al. 2022; Mavian et al. 2023; Faruque et al. 2005). These environmental surveillance studies could simultaneously provide information on additional topics central to cholera control, including information on *V. cholerae* drug resistance.

Furthermore, if so determined that pandemic *V. cholerae* does not form long‐term reservoirs, further exploration is needed to understand why, when it appears other *V. cholerae* strains and *Vibrio* species do, a question unanswered even by studies refuting the reservoir hypothesis. One co‐author has previously hypothesized pathogenicity factors like toxin‐coregulated pilus, which improve the bacteria's ability to infect humans, may somehow result in decreased environmental fitness compared to other *Vibrios*. However, while supported by the absence of the genetic island for this pilus in many non‐pandemic *V. cholerae* strains, this theory has not been tested (Mekalanos et al. 1997). Studies on the role of both bacterial elements and other ecological factors in environmental persistence could provide evidence in favour of or against the plausibility and mechanism of long‐term reservoirs. For example, water samples could also be tested for bacteria such as *Aeromonas dhakensis* strain A603—which has demonstrated vibriocidal activity—to explore whether changes in predatory bacterial population sizes correlate with changes in cholera rates, as has been demonstrated with predatory phages (Faruque et al. 2005; Bier et al. 2024).

Second, the role of inapparent infection in overall cholera transmission requires further understanding. Studies have documented infections with *V. cholerae* resulting in few or no symptoms, but multiple outstanding questions remain, from the infectiousness of people with inapparent infection (compared to patients with classic ‘rice water’ stool) to the patient‐level factors predisposing to severe versus inapparent disease, to how rates of inapparent infection may differ between cholera‐endemic regions and places experiencing less frequent outbreaks (Hegde et al. 2024). These knowledge gaps can be addressed by larger‐scale human surveillance studies to include identification of asymptomatic or subclinical carriers among geographically diverse populations. If inapparent infection is found to be common across geographic regions, this would strengthen its case over the environmental reservoir hypothesis to explain trends in Haiti and elsewhere.

Inadequate progress towards global water, sanitation and hygiene goals, a critical lack of human resources for health and health infrastructure, desperate poverty, as well as other factors, mean that billions of people remain at risk of cholera in 2026 and beyond. Despite much progress, serious gaps in knowledge persist which hamper an evidence‐based approach to prediction, prevention and control of the disease. We propose that a better understanding of the role of the environment and human factors on the persistence and recurrence of cholera should be elevated as priorities (World Health Organization 2025).

## Author Contributions

**Azfar D. Hossain:** conceptualization, writing – original draft, writing – review and editing, visualization. **William P. Robins:** writing – review and editing. **John J. Mekalanos:** writing – review and editing. **Louise C. Ivers:** conceptualization, writing – review and editing, supervision.

## Conflicts of Interest

The authors declare no conflicts of interest.

## Data Availability

The authors have nothing to report.
